# The Role of Placental Mitochondrial Dysfunction in Adverse Perinatal Outcomes: A Systematic Review

**DOI:** 10.3390/jcm14113838

**Published:** 2025-05-29

**Authors:** Charalampos Voros, Sofoklis Stavros, Ioakeim Sapantzoglou, Despoina Mavrogianni, Maria Anastasia Daskalaki, Marianna Theodora, Panagiotis Antsaklis, Peter Drakakis, Dimitrios Loutradis, Georgios Daskalakis

**Affiliations:** 1 1st Department of Obstetrics and Gynecology, ‘Alexandra’ General Hospital, National and Kapodistrian University of Athens, 80 Vasilissis Sofias Avenue, 11528 Athens, Greece; kimsap1990@hotmail.com (I.S.); depy.mavrogianni@yahoo.com (D.M.); md181341@students.euc.ac.cy (M.A.D.); martheodr@gmail.com (M.T.); panosant@gmail.com (P.A.); gdaskalakis@yahoo.com (G.D.); 2 3rd Department of Obstetrics and Gynecology, Attikon Hospital, National and Kapodistrian University of Athens, Rimini 1, 12462 Chaidari, Greece; pdrakakis@hotmail.com; 3 Fertility Institute-Assisted Reproduction Unit, Paster 15, 11528 Athens, Greece; loutradi@otenet.gr; 4 Athens Medical School, National and Kapodistrian University of Athens, 15772 Athens, Greece

**Keywords:** placenta, mitochondrial dysfunction, preeclampsia, IUGR, perinatal outcomes, oxidative stress, systematic review

## Abstract

**Background:** Mitochondria are essential for placental function as they regulate energy metabolism, oxidative balance, and apoptotic signaling. Increasing evidence suggests that placental mitochondrial dysfunction may play a role in the development of many poor perinatal outcomes, including preeclampsia, intrauterine growth restriction (IUGR), premature birth, and stillbirth. Nonetheless, no systematic review has thoroughly investigated this connection across human research. This study aims to consolidate evidence from human research concerning the link between placental mitochondrial dysfunction and negative birth outcomes. **Methods**: A systematic search of PubMed, Scopus, and Web of Science identified human research examining placental mitochondrial features (e.g., mtDNA copy number, ATP production, oxidative stress indicators) in connection with adverse pregnancy outcomes. Methodological variety resulted in narrative data extraction and synthesis. **Results**: Twenty-nine studies met the inclusion criteria. Mitochondrial dysfunction was consistently associated with PE, IUGR, FGR, and PTB. The most often observed outcomes included diminished mtDNA copy number, decreased ATP production, elevated reactive oxygen species (ROS), and disrupted mitochondrial dynamics, characterized by increased DRP1 and decreased MFN2. Early-onset preeclampsia and symmetric fetal growth restriction exhibited particularly severe mitochondrial abnormalities, indicating a primary placental origin of the condition. **Conclusions**: A significant factor contributing to adverse pregnancy outcomes is the dysfunction of placental mitochondria. The analogous molecular signatures across many disorders suggest promising avenues for developing targeted therapies aimed at improving maternal–fetal health and predictive biomarkers.

## 1. Introduction

Mitochondria are critical organelles that control a variety of cellular processes, such as energy generation via oxidative phosphorylation, redox equilibrium maintenance, calcium signaling, and apoptotic pathway regulation [1]. Their importance goes beyond bioenergetics and includes important contributions to cell survival, differentiation, and stress responses. In the human placenta, mitochondria are numerous in both the syncytiotrophoblast and cytotrophoblast layers, where they help to move nutrients, gases, and metabolic waste between the maternal and fetal circulations [2]. Placental mitochondria maintain the structural integrity and functional competence of trophoblast cells, which are required for proper fetal development, by producing adenosine triphosphate (ATP) and regulating reactive oxygen species (ROS) levels [3].

Given the placenta’s distinct and dynamic environment, which includes rapidly changing oxygen levels, hormonal influences, and immune interactions, the mitochondrial population must be highly plastic, adapting through tightly regulated processes such as mitochondrial biogenesis, fusion and fission dynamics, and mitophagy [4]. These alterations are not exclusive to preeclampsia but are also characteristic of other pregnancy problems, such as gestational diabetes mellitus (GDM), in which both placental and maternal serum exhibit increased oxidative stress, mitochondrial fragmentation, and compromised bioenergetics [5,6,7]. These mitochondrial disruptions have been linked to the pathophysiology of various obstetric problems, highlighting the placenta’s susceptibility to mitochondrial dysregulation [8].

Emerging research suggests that disruptions in placental mitochondrial bioenergetics, biogenesis, dynamics, and oxidative stress responses are key factors causing poor neonatal outcomes [9]. Aberrant mitochondrial activity has been linked to insufficient trophoblast invasion, defective vascular remodeling, and placental insufficiency, all of which are frequent in pregnancy issues such preeclampsia, intrauterine growth restriction (IUGR), premature delivery, and stillbirth [10]. Furthermore, changes in mitochondrial DNA (mtDNA) copy numbers, expression of mitochondrial respiratory chain complexes, and oxidative damage markers have been consistently observed in placental tissues from complicated pregnancies, implying a direct relationship between mitochondrial health and pregnancy success [11]. As mentioned already, aberrant placental mitochondrial activity is thought to directly cause negative pregnancy outcomes [4]. One of the most often reported changes is a disruption of mtDNA integrity and copy number. Deletions, mutations, or decreases in mtDNA copy numbers indicate reduced mitochondrial biogenesis, whereas increasing mtDNA content might be a compensatory response to increased oxidative stress [12].

In addition to mtDNA modifications, diseased placentas have shown large reductions in adenosine triphosphate (ATP) generation, indicating faulty oxidative phosphorylation along the electron transport chain (ETC) [13]. Dysregulation of critical ETC complexes, notably complexes I and IV, impairs electron transport, encouraging electron leakage and the overproduction of reactive oxygen species (ROS). Elevated ROS levels damage mitochondrial components and activate redox-sensitive signaling pathways including NF-κB and HIF-1α, aggravating inflammation, hypoxia, and vascular dysfunction in the placenta [14]. Furthermore, the equilibrium between mitochondrial fusion and fission, which is essential for mitochondrial integrity, is frequently altered in unfavorable pregnancies. Reduced expression of fusion mediators such as Mitofusin 1 (MFN1), Mitofusin 2 (MFN2), and Optic atrophy protein 1 (OPA1), combined with an increase or hyperactivation of fission-related proteins such as Dynamin-related protein 1 (DRP1) and Fission 1 (FIS1), causes mitochondrial fragmentation, membrane potential loss, and impaired bioenergetic function [15]. These modifications impair mitochondria’s capacity to react to stresses and maintain trophoblast viability during the dynamic circumstances of gestation.

In addition, complex pregnancies affect mitochondrial quality control mechanisms, including mitophagy. Dysregulation of mitophagy mediators, such as PTEN-induced kinase 1 (PINK1) and Parkin (PARK2), leads to impaired clearance of damaged mitochondria, the buildup of malfunctioning organelles, and increased cellular stress. Defects in mitochondrial unfolded protein response (UPRmt) components, such as caseinolytic peptidase B protein homolog (CLPP), have also been linked, implying a larger impairment of mitochondrial proteostasis [16].

At a larger level, mitochondrial malfunction in the placenta has been associated with aberrant angiogenesis, altered vasoactive factor synthesis, and impaired immunomodulatory activities, all of which are required for proper placental growth and fetal maintenance. For example, altered mitochondrial activity might result in decreased generation of nitric oxide (NO), a chemical required for vascular tone modulation, leading to the endothelial dysfunction seen in preeclampsia [17].

Understanding mitochondrial changes at the placental level holds great potential for developing diagnostic and therapeutic techniques to improve mother and fetal health. Because mitochondria are critical regulators of placental energy metabolism, oxidative balance, and cellular survival, even minor dysfunctions can have a significant impact on trophoblast differentiation, invasion, and vascular remodeling—all critical processes for successful pregnancy establishment and maintenance [18]. Identifying mitochondrial patterns linked with abnormal pregnancies may give early illness prediction indicators, allowing doctors to stratify risk and act before clinical symptoms appear.

Molecular analysis of placental tissue has already identified promising indicators. Variations in mitochondrial DNA (mtDNA) copy number, accumulation of oxidative stress markers such as 8-hydroxy-2′-deoxyguanosine (8-OHdG) and malondialdehyde (MDA), and altered expression of fusion–fission proteins such as Mitofusin 2 (MFN2) and Dynamin-related protein 1 (DRP1) have all been proposed as indicators of placental mitochondrial function [19]. Furthermore, downregulation of mitochondrial quality control proteins such as PTEN-induced kinase 1 (PINK1) and Parkin (PARK2), as well as changes in the mitochondrial unfolded protein response mediated by caseinolytic peptidase B protein homolog (CLPP), demonstrate the utility of molecular markers reflecting mitochondrial stress or failure [20].

Beyond diagnostics, understanding the underlying molecular processes that link mitochondrial dysfunction to poor pregnancy outcomes may pave the way for tailored treatments. Antioxidants, mitochondrial biogenesis activators, and mitochondrial dynamics modulators are examples of therapeutic approaches that may prevent or improve disorders such as preeclampsia and intrauterine growth restriction [21]. Furthermore, developing techniques including mitochondrial transplantation, gene therapy targeting mitochondrial regulators, and manipulation of mitophagy pathways are being intensively researched in other domains and may someday be used to obstetric care [22]. Despite theoretical potential, many problems persist. The placenta is a very diverse and dynamic organ, with mitochondrial activity altering depending on cell type, gestational age, and pathogenic setting [9]. As a result, future research must precisely define mitochondrial changes in a cell-specific and time-resolved way, combine multi-omic techniques, and verify prospective biomarkers and therapeutics through rigorous clinical trials [23]. Bridging the gap between molecular findings and clinical application will be critical for realizing the full potential of placental mitochondrial biology in maternal–fetal care.

The goal of this systematic review is to compile and critically evaluate the available human data associating placental mitochondrial dysfunction to poor neonatal outcomes. Particular emphasis will be placed on identifying recurrent molecular signatures, such as altered expression of fusion and fission proteins (e.g., Mitofusin 1/2 [MFN1/2], Dynamin-related protein 1 [DRP1]), dysregulation of mitophagy mediators (PTEN-induced kinase 1 [PINK1] and Parkin [PARK2]), and markers of oxidative stress damage, such as 8-hydroxy-2′-deoxyguanosine (8-OHdG) and malondialdehyde (MDA). This study aims to understand the molecular processes behind placental insufficiency while also identifying promising biomarkers for early identification and therapeutic intervention. Furthermore, by emphasizing methodological variation between studies—such as differences in mitochondrial assessment procedures, gestational timing, and outcome definitions—this review seeks to identify current research gaps and propose standardized approaches for future studies. Finally, a better understanding of placental mitochondrial dysfunction has the potential to transform high-risk pregnancy management, paving the way for more precise, mechanism-based approaches for preventing or mitigating preeclampsia, intrauterine growth restriction (IUGR), preterm birth, and stillbirths.

## 2. Material and Methods

This systematic review adhered to the preferred reporting items for systematic reviews and meta-analyses (PRISMA) 2020 standards. The review methodology was recorded prospectively in the international prospective register of systematic reviews (PROSPERO) with the ID CRD420251028536.

### 2.1. Literature Search Strategy

A complete literature search was carried out in the following three main electronic databases: PubMed, Scopus, and Web of Science. The search method included keywords and medical subject heading (MeSH) terminology linked to placental mitochondrial dysfunction and poor perinatal outcomes. A number of phrases were used: “placenta”, “mitochondrial dysfunction”, “mitochondrial DNA”, “oxidative stress”, “preeclampsia”, “intrauterine growth restriction”, “preterm birth”, and even “stillbirth”. The search was limited to human research published in English until April 2025. There were no criteria based on study design or publication type. Table 1 shows the precise search technique for each database.

The search algorithms were created to locate research that investigated placental mitochondrial dysfunction in connection with unfavorable neonatal outcomes. Boolean operators (AND, OR) and MeSH keywords (where appropriate) were utilized to improve the sensitivity and specificity of the searches across all three databases. Except for restricting findings to human studies published in English, no language, publication date, or research design constraints were imposed at the start.

To achieve a thorough and systematic identification of relevant research, a sophisticated search strategy was used across the following three main electronic databases: PubMed, Scopus, and Web of Science.

The search method was carefully designed to maximize the sensitivity and specificity of the returned literature by combining the medical subject heading (MeSH) when appropriate, free-text keywords, and Boolean operators (“AND”, “OR”).

In PubMed, the strategy combined controlled vocabulary terms (MeSH) such as “placenta”, “mitochondria”, and “oxidative stress”, with keyword variations such as “mitochondrial dysfunction” and relevant perinatal outcomes (“preeclampsia”, “intrauterine growth restriction”, “preterm birth”, and “stillbirth”). This dual strategy guaranteed the inclusion of studies that had not yet been indexed with MeSH keywords but were highly relevant based on title or abstract content. In Scopus and Web of Science, whose standardized indexing techniques differ from PubMed, the search was performed utilizing title, abstract, and keyword fields. The logical combination of keywords reflected PubMed’s technique for maintaining uniformity across databases.

The search was purposely wide in order to include as many observational studies as possible investigating mitochondrial characteristics in the human placenta that are associated with poor pregnancy outcomes. There were no initial limits on the research design or publication date. However, only research published in English and conducted on human populations were considered suitable throughout the screening process. Table 1 provides a detailed analysis of the precise search words used in each database, demonstrating search repeatability and transparency in compliance with PRISMA 2020 reporting criteria.

### 2.2. Eligibility Criteria

Studies were deemed eligible if they studied human pregnancies, including singleton or multiple gestations, and directly assessed placental mitochondrial characteristics. Eligible parameters included mtDNA content, ATP production, ROS levels, oxidative stress markers, indicators of mitochondrial biogenesis, and regulators of mitochondrial dynamics and quality control, such as fusion and fission proteins, mitophagy components, and the mitochondrial unfolded protein response (UPRmt).

To be included, studies had to disclose at least one poor perinatal outcome associated with mitochondrial dysfunction, namely PE (preeclampsia), IUGR, PTB (preterm birth), or FGR (fetal growth restriction). The investigation of mitochondrial function was required to be performed on placental tissue samples to ensure biological relevance to the maternal–fetal interface. Observational study designs, such as case–control, cohort, and cross-sectional studies, were considered appropriate, as were experimental investigations including human placental samples, as long as mitochondrial activity was quantitatively or subjectively measured. Studies that used only animal models, ex vivo systems, or in vitro experimental approaches were omitted. Furthermore, research was omitted if mitochondrial function was not directly addressed or if no particular prenatal clinical outcomes were recorded, such as those that focused primarily on mechanistic cellular pathways with little clinical relevance.

There were no constraints on the gestational age at sample, the method utilized for mitochondrial evaluation (e.g., mtDNA quantification, ATP measurement, ATPtests, immunohistochemistry, Western blotting, or transcriptome studies), or the severity of the adverse event. However, only research published in English and involving human subjects were considered eligible to assure the retrieved data’s consistency and applicability. The adoption of these qualifying criteria enabled the targeted selection of high-relevance studies that directly addressed the link between placental mitochondrial dysfunction and unfavorable pregnancy outcomes in human clinical populations.

### 2.3. Study Selection

After the database searches were completed, all detected records were transferred into reference management software, where duplicates were carefully deleted. Two reviewers separately reviewed the titles and abstracts of the retrieved publications for relevance to placental mitochondrial dysfunction and unfavorable perinatal outcomes, especially PE, IUGR, PTB, and FGR.

During the initial screening phase, articles that plainly did not match the inclusion requirements were rejected, including non-human research, reviews, editorials, and studies unrelated to mitochondrial function. Full-text publications from potentially eligible studies were then obtained and rigorously screened for ultimate inclusion eligibility. Both reviewers independently reviewed the whole text to establish if the research directly measured mitochondrial characteristics such as mtDNA content, ATP generation, ROS levels, mitochondrial biogenesis, dynamics, or quality control, and whether they reported relevant clinical outcomes.

**Figure 1:**
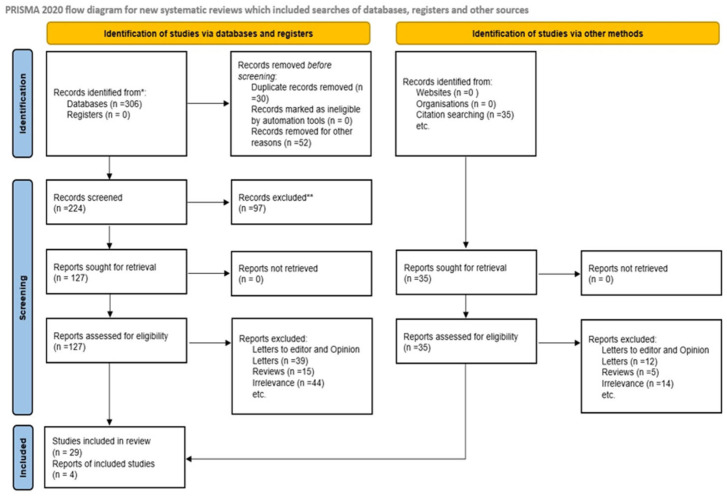
Flow diagram. * Consider, if feasible to do so, reporting the number of records identified from each database or register searched (rather than the total number across all databases/registers). ** If automation tools were used, indicate how many records were excluded by a human and how many were excluded by automation tools.

Disagreements between reviewers throughout the title/abstract screening and full-text review stages were addressed by discussion and, if required, contact with a third reviewer. There were no automated technologies or artificial intelligence-assisted selection methods employed throughout the screening process to ensure human judgment and correctness. Figure 1 depicts the research selection process, including the number of studies identified, screened, eliminated, and included in the final qualitative synthesis, following the PRISMA 2020 flow diagram. This stringent screening approach enabled the inclusion of high-quality research that directly addressed the link between placental mitochondrial dysfunction and poor neonatal outcomes in human populations.

### 2.4. Data Extraction

Two reviewers extracted data separately, using a pre-specified standardized data collecting form, to guarantee methodological rigor and reduce extraction mistakes. Bibliographic information about each included study was recorded, including the initial author’s name, the year of publication, and the nation or geographical location where the study was conducted. The study design was classified as case–control, cohort, or cross-sectional based on the authors’ description and methodological characteristics. Regarding clinical data, the unfavorable prenatal outcomes investigated—namely PE, IUGR, PTB, and FGR—were meticulously documented. Each outcome utilized in the research was defined specifically, taking into account potential variation in clinical diagnostic criteria among groups.

Comprehensive extraction of mitochondrial parameters was performed. These included quantitative measurements of mtDNA content or integrity, ATP production levels, ROS generation, and mitochondrial biogenesis and dynamics. The expression levels of fusion-related proteins like MFN1 and MFN2, as well as fission regulators like DRP1, were extracted. Furthermore, data on mitochondrial quality control mechanisms, namely the role of PINK1, PARK2, and CLPP, were extensively gathered. When oxidative stress indicators, such as MDA and 8-OHdG, were reported, they were noted with any other biochemical or molecular markers of mitochondrial health.

The methodological approaches used in each study to evaluate mitochondrial function—such as quantitative PCR techniques, Western blotting, immunohistochemistry, and specific oxidative stress assays—were also documented to aid in the interpretation of methodological variability across the included studies. Throughout the extraction process, disagreements amongst reviewers were resolved by debate and consensus. When consensus could not be reached, a third reviewer was consulted to settle issues. To preserve the accuracy of human judgment, no automated technologies were employed throughout the data extraction process.

Table 2 summarizes the general methodological aspects as well as the significant findings from the included research.

To improve the clarity, openness, and interpretability of the synthesis information, a summary of findings table (Table 2) was developed. This table presents a systematic summary of the key mitochondrial results reported in the included research, with an emphasis on molecular and functional changes discovered in placental tissues related to poor neonatal outcomes. For each investigation, the following data were thoroughly gathered and summarized: (1) the adverse pregnancy outcome under investigation, such as PE, IUGR, preterm birth (PTB), and fetal growth restriction (FGR); and (2) the specific mitochondrial parameters evaluated, such as mtDNA content or integrity, ATP production levels, ROS generation, and mitochondrial dynamics regulators, such as Mitofusin 1 (MFN1), Mitofusin 2 (MFN2), and DRP1.

The examination also covered mitochondrial quality control mechanisms, including PTEN-induced kinase 1 (PINK1), Parkin (PARK2), and caseinolytic peptidase B homolog (CLPP), which regulate mitophagy and the mitochondrial unfolded protein response (UPRmt). Oxidative stress indicators such as malondialdehyde (MDA) and 8-hydroxy-2′-deoxyguanosine (8-OHdG) were also isolated and reported. Each study’s major finding on the involvement of mitochondrial dysfunction in the reported unfavorable outcome was noted to provide a succinct yet informative summary. This synthesis allows for the detection of recurrent mitochondrial problems such as decreased mtDNA copy number, poor ATP generation, excessive ROS buildup, changed mitochondrial biogenesis, and disturbed fusion–fission balance in a variety of clinical situations. By aggregating findings from various study designs and populations, the summary of findings table allows for direct comparison, highlights patterns in mitochondrial dysfunction associated with specific outcomes, and aids in the identification of potential molecular biomarkers or therapeutic targets. Furthermore, providing the data in a uniform and structured manner enhances the repeatability and transparency of this systematic review, as per the PRISMA 2020 standards.

### 2.5. Risk of Bias Assessment

The methodological quality of the included studies was thoroughly examined by two independent reviewers who used the Newcastle–Ottawa scale (NOS), a widely acknowledged methodology for assessing observational studies in systematic reviews and meta-analysis. The NOS examines the risk of bias in three essential domains, research group selection, case–control comparability, and exposure or outcome determination, depending on the study design.

Each included study was rigorously appraised using specific NOS criteria. The “Selection” domain determined if the cases were well-characterized and represented, as well as whether the controls were chosen effectively. The “Comparability” domain assessed the extent to which possible confounders were addressed during the research design or analysis phase. The “Exposure” (for case–control studies) or “Outcome” (for cohort and cross-sectional studies) domain evaluated the robustness of the methods used to determine mitochondrial parameters such as mtDNA content, ATP production, ROS levels, and markers related to mitochondrial biogenesis, dynamics, and quality control, as well as the validity of clinical perinatal outcomes reported, such as PE, IUGR, PTB, and FGR.

Each domain was rated, in Table 3, as “Good”, “Moderate”, or “Poor” based on predetermined criteria that represent methodological rigor. Studies that matched the criteria in all dimensions were classed as “low risk of bias”, whereas those with slight methodological problems were classified as “moderate risk of bias”. Studies with major faults impacting internal validity would have been designated “high risk of bias”, however no study was categorized in this category. Disagreements among reviewers were addressed by discussion and consensus. Table 2 summarizes the total risk of bias assessment and provides a comprehensive review of the methodological strengths and flaws of the included studies. This rigorous assessment ensures the trustworthiness and interpretability of the evidence gathered in this systematic review.

### 2.6. Summary of Findings

To allow for an organized and visible synthesis of the retrieved information, a summary of findings table was created, which presented the key mitochondrial abnormalities identified in each included study. Every study reported the poor perinatal outcomes measured, such as PE, IUGR, PTB, and FGR, in addition to the critical mitochondrial characteristics studied. The mitochondrial characteristics summarized included changes in mtDNA content or integrity, ATP generation, ROS levels, and the expression of mitochondrial dynamics regulators MFN1, MFN2, and DRP1. In addition, observations on mitochondrial quality control proteins such as PINK1, PARK2, and CLPP were published, as well as measures of oxidative stress indicators such as MDA and 8-OHdG.

Each study’s main result on the link between mitochondrial dysfunction and poor perinatal outcomes was briefly described to enable quick comparison across the body of data. The table identifies recurrent molecular abnormalities—such as decreased mtDNA copy number, reduced ATP generation, higher ROS levels, dysregulation of mitochondrial biogenesis, and imbalances in mitochondrial fusion and fission—that have been linked to various clinical settings. By structuring the retrieved data in a systematic manner, the summary of findings table allows for the discovery of consistent mitochondrial signatures that may be implicated in the pathophysiology of unfavorable pregnancy outcomes. Furthermore, it improves the review’s repeatability and transparency, in line with the PRISMA 2020 principles. Table 4 presents the complete findings.

## 3. Results

### 3.1. Initial Results

The initial results of the systematic search across the identified databases were 306 documents. After removing 30 duplicate entries and excluding 52 records for various reasons, such as inappropriate article kinds or insufficient data, there were 224 unique records left for title and abstract screening. Two reviewers completed the preliminary screening procedure separately, and any conflicts were handled by consensus discussion. During the title and abstract screening step, 97 records were removed. The bulk of these exclusions were due to a lack of relevance to placental mitochondrial dysfunction, an absence of measurement of mitochondrial markers such as mtDNA, ATP, and ROS, or a failure to disclose clinical outcomes associated with unfavorable perinatal events such as PE, IUGR, PTB, and FGR.

After the first screening, 127 full-text publications were identified and evaluated for eligibility. After full-text screening, 98 articles were excluded. Specifically, 39 reports were letters to the editor or opinion articles that lacked original research data, 15 were review articles that did not present primary study data, and 44 were excluded for being irrelevant, either because they did not assess mitochondrial function in placental tissues or because they failed to report associations with clinical outcomes. In parallel, more studies were discovered through citation searching, generating 35 new records. All of these records were subjected to full-text analysis, and 31 were removed for reasons identical to those used in database searches as 12 were letters or opinion articles, 5 were reviews, and 14 were unrelated to the study question.

The final qualitative synthesis included 29 papers that fulfilled the inclusion criteria. These studies looked primarily at mitochondrial activity in human placental tissues in the setting of poor perinatal outcomes. Figure 1 depicts the whole study selection process, including the number of records identified, vetted, eliminated, and included.

### 3.2. Study Characteristics

This comprehensive review includes 29 papers with significant variety in research design, demographic characteristics, placental sample procedures, mitochondrial parameters examined, and clinical outcomes explored.

The majority of the included studies used a case–control strategy, comparing placental mitochondrial characteristics between pregnancies complicated by unfavorable outcomes such as PE, IUGR, PTB, or FGR and healthy, normotensive control pregnancies. A smaller minority used prospective cohort designs, which tracked pregnancies over time to assess the onset of unfavorable outcomes related to placental mitochondrial function. Few studies used cross-sectional methods, focusing on placental samples taken at the moment of delivery. The investigations included a wide range of geographical areas, including North America (the United States and Canada), Europe (the United Kingdom, France, Germany, and Italy), Asia (China, Japan, and India), and Australia. This worldwide distribution increased the findings’ generalizability while also introducing diversity due to clinical practice patterns, diagnostic criteria for PE, IUGR, PTB, and FGR, and population demographics such as maternal age, ethnicity, and comorbidities.

The sample sizes in the included research varied greatly, ranging from small studies with less than 20 subjects per group to bigger investigations with over 150 placental samples. To account for possible confounders such as chorionicity and twin-specific problems, most studies only looked at singleton pregnancies. However, a few studies focused especially on mitochondrial malfunction in multiple pregnancies, particularly selective FGR in monochorionic twin gestations. PE was the most commonly studied condition for unfavorable perinatal outcomes, followed by IUGR, FGR, and PTB. Several investigations distinguished between early-onset and late-onset PE, with early-onset cases being linked with more severe mitochondrial abnormalities. Similarly, other research distinguished between symmetric and asymmetric versions of FGR. The definition of unfavorable outcomes was typically based on known clinical criteria; however, there was significant variation in diagnostic thresholds and categorization methods between investigations.

The mitochondrial parameters tested varied greatly, but they all contained important indications of mitochondrial health and function. Quantitative assessments of mtDNA copy number were prevalent, indicating a focus on mitochondrial content as an indicator of placental stress or malfunction. Several studies examined ATP production levels to determine the placenta’s bioenergetic capability. ROS production and oxidative stress were regularly measured, with a focus on indicators like MDA and 8-OHdG. A collection of research focused on mitochondrial dynamics, assessing the expression of fusion-promoting proteins including MFN1 and MFN2, as well as fission mediators like DRP1. Many of the mitochondrial abnormalities reported in impaired pregnancies were thought to be caused by disruptions in the balance of mitochondrial fusion and fission.

Furthermore, other research looked at mitochondrial quality control mechanisms, including the expression and activity of proteins including PINK1, PARK2, and CLPP, which regulate mitophagy and mitochondrial integrity under stress. Alterations in UPRmt were also investigated as indications of mitochondrial dysfunction. Various molecular and biochemical approaches were used to evaluate mitochondrial characteristics. Quantitative PCR was the most popular approach for determining mtDNA content. Western blotting and immunohistochemistry were commonly utilized to analyze protein expression associated with mitochondrial dynamics and oxidative stress. Some research used high-resolution respirometry to directly evaluate mitochondrial respiratory function, whilst others used ELISA-based techniques to identify oxidative stress indicators. Data extraction and compilation took into account the variability of methodological techniques, patient demographics, and outcome definitions among the studies. These distinctions are crucial to consider when understanding the overall findings, and they will be addressed in the narrative synthesis.

A summary of findings table (Table 4) is included to enhance the clarity, consistency, and interpretability of the results. This table provides a comprehensive and uniform overview of the evidence base by consolidating essential methodological components, inclusion and exclusion criteria, evaluated results, and analyzed mitochondrial parameters from each included study.

Table 4 summarizes the major mitochondrial changes identified in the included studies. Overall, there was a consistent pattern of placental mitochondrial dysfunction associated with unfavorable perinatal outcomes such as PE, IUGR, PTB, and FGR. A consistent and notable finding across studies was a considerable drop in mtDNA copy number in placental tissues impacted by unfavorable outcomes. Decreased mtDNA content was especially noticeable in cases of early-onset PE and severe types of IUGR, indicating decreased mitochondrial biogenesis or accelerated mitochondrial turnover in response to placental stress. Several studies have connected reduced mtDNA copy number to impaired mitochondrial respiratory activity and energy deficiency, supporting the idea that mitochondrial depletion may impair the ability of a placenta to sustain fetal development and maternal homeostasis. Reduced ATP generation was commonly seen in conjunction with mtDNA depletion. Decreased ATP levels are a direct result of decreased oxidative phosphorylation and mitochondrial malfunction, underlining the placenta’s bioenergetic failure in complex pregnancies. These bioenergetic deficiencies are especially essential in the placenta, which has high energy requirements to maintain active transport systems and hormone production. A rise in oxidative stress was another significant result. Elevated ROS levels, as well as elevated indicators of oxidative damage such as MDA and 8-OHdG, were linked to a variety of negative consequences. Oxidative stress appears to be both a cause and a consequence of mitochondrial malfunction, resulting in a vicious cycle of mitochondrial injury, poor energy generation, and increasing cellular damage inside the placenta.

Alterations in mitochondrial dynamics were also commonly reported. Several investigations found a shift toward enhanced mitochondrial fission, as shown by an overexpression of DRP1 and a decreased expression of fusion-related proteins such MFN1 and MFN2. The imbalance between mitochondrial fusion and fission causes excessive mitochondrial fragmentation, loss of mitochondrial network integrity, and functional degradation. Such fragmentation has been linked to PE etiology, where mitochondrial structural abnormalities are evident in histological investigations. In addition, some investigations found abnormalities in mitochondrial quality control pathways, particularly PINK1, PARK2, and CLPP. Impaired mitophagy causes a buildup of malfunctioning mitochondria, which exacerbates oxidative stress and bioenergetic failure. The activation of the UPRmt was inconsistently reported; whereas some studies found upregulation as a compensatory mechanism, others found downregulation, implying an overwhelmed or exhausted mitochondrial stress response in extreme situations.

Notably, whereas the overall pattern of mitochondrial dysfunction was similar across PE, IUGR, PTB, and FGR, there were minor variances. For example, in early-onset PE, mitochondrial oxidative damage and energy deficiencies were more severe than in late-onset PE, indicating that placental mitochondrial pathology plays a larger role in more severe clinical symptoms. Similarly, in cases of selective FGR in twin pregnancies, mitochondrial failure was limited to the smaller twin’s placenta, highlighting the clear relationship between mitochondrial health and fetal growth outcomes. Table 4 summarizes these data, emphasizing the critical role of mitochondrial dysfunction in the pathophysiology of a wide range of poor neonatal outcomes. It identifies important molecular pathways—such as mtDNA depletion, decreased ATP synthesis, oxidative stress, mitochondrial dynamics imbalance, and poor quality control—that might serve as diagnostic or therapeutic targets in the future management of complex pregnancies.

### 3.3. Subgroup Analyses

#### 3.3.1. Preeclampsia

Several included studies classified PE patients based on gestational age upon diagnosis, distinguishing between early-onset and late-onset PE, with a usual threshold of 34 weeks of gestation. Early-onset PE was consistently linked with more severe mitochondrial dysfunction than late-onset PE, indicating that placental mitochondrial abnormalities play a more basic role in the etiology of early disease types.

Reductions in mtDNA copy number were much more evident in early-onset PE, indicating defective mitochondrial biosynthesis or accelerated mitochondrial degradation processes during the early phases of placental development. In parallel, early-onset patients showed a significant reduction in ATP synthesis, indicating serious bioenergetic failure. These abnormalities represent a fundamental impairment of the placenta’s capacity to satisfy the metabolic needs necessary for healthy embryonic development and maternal adaptation during pregnancy.

Another trait that distinguished early- versus late-onset PE was oxidative stress. Studies consistently found greater levels of ROS, MDA, and 8-OHdG in early-onset cases, indicating an increased oxidative load in the placenta. This increased oxidative stress most likely exacerbates mitochondrial damage, contributing to a self-sustaining loop of mitochondrial malfunction, tissue injury, and inflammatory response, all of which have been linked to PE pathogenesis. Furthermore, variations in mitochondrial dynamics were observed between early- and late-onset illness. Early-onset PE was related with a considerable overexpression of DRP1, which promotes mitochondrial fission, and a downregulation of fusion-promoting proteins including MFN1 and MFN2. This imbalance causes excessive mitochondrial fragmentation, which disrupts mitochondrial networks, reduces respiratory efficiency, and increases vulnerability to mitochondria-mediated apoptosis. Such fragmentation has been observed histologically in placental tissues from early-onset PE, supporting the molecular results.

Importantly, alterations in mitochondrial quality control mechanisms were more commonly observed in early-onset PE. Several studies found a lower expression of PINK1 and PARK2, implying defective mitophagy and an insufficient clearance of damaged mitochondria. Failure to clear malfunctioning mitochondria likely exacerbates oxidative stress, bioenergetic insufficiency, and placental abnormalities. Late-onset PE, on the other hand, was linked with lesser alterations, despite the fact that some mitochondrial modification occurred. Reductions in mtDNA content and ATP generation were less severe, although oxidative stress indicators were substantially enhanced when compared to normotensive controls. This shows that in late-onset PE, maternal variables such as pre-existing cardiovascular risk or metabolic disorders may play a larger role in disease development, whereas placental mitochondrial dysfunction is more important in early-onset instances.

#### 3.3.2. Intrauterine Growth Restriction and Fetal Growth Restriction

Placental mitochondrial malfunction was a common observation in pregnancies complicated by IUGR and FGR, emphasizing the importance of mitochondrial integrity in promoting normal fetal development. Molecular analysis from numerous studies revealed significant changes in critical mitochondrial pathways, indicating an important role for defective mitochondrial metabolism and quality control in the etiology of growth limitation. A substantial drop in the mtDNA copy number was seen in placentas from IUGR/FGR pregnancies when compared to controls. This decrease indicates either a failure in mitochondrial biogenesis or an increase in mitochondrial breakdown through mitophagy. Given that placental growth and function rely largely on a sufficient mitochondrial population to fulfill high metabolic demands, mtDNA decreases can directly impair the placenta’s energy generation capacities.

ATP generation was also significantly decreased in IUGR/FGR patients, indicating defective oxidative phosphorylation. This bioenergetic failure is likely caused by a combination of mtDNA depletion and direct damage to mitochondrial respiratory chain complexes. Disruptions in complexes I, III, and IV of the electron transport chain have been linked to inefficient ATP synthesis and increased electron leakage, both of which contribute to ROS formation. Elevated ROS production was a common molecular hallmark in IUGR/FGR placenta. Oxidative stress destroys mitochondrial DNA, lipids, and proteins, resulting in a self-perpetuating cycle of mitochondrial injury. Increased levels of MDA and 8-OHdG were regularly found, indicating lipid peroxidation and oxidative DNA damage, respectively. Sustained oxidative stress activates pro-apoptotic pathways including cytochrome-c release, caspase activation, and mitochondrial-mediated apoptosis, resulting in trophoblast cell loss and placental insufficiency.

Analysis of mitochondrial dynamics indicated a significant trend towards mitochondrial fragmentation. Studies showed that DRP1 was upregulated whereas MFN1 and MFN2 were downregulated, indicating a fission-dominated phenotype. Mitochondrial fragmentation not only reduces mitochondrial energy efficiency but it also prepares mitochondria for mitophagy or apoptotic signaling. Excessive mitochondrial fission has been associated with decreased syncytiotrophoblast production and function, which worsens placental insufficiency. Aside from mitochondrial dynamics, considerable changes in mitochondrial quality-control systems were detected. Several studies have reported dysregulation of PINK1–PARK2-mediated mitophagy. PINK1 normally accumulates on the outer mitochondrial membrane in response to mitochondrial depolarization, where it recruits PARK2 to trigger the selective destruction of injured mitochondria. However, in IUGR/FGR placentas, decreased expression or function of these proteins was described, resulting in the buildup of defective mitochondria and perpetuation of oxidative stress.

Furthermore, research shows that the UPRmt pathway, a mitochondrial-specific stress response meant to restore proteostasis inside mitochondria, is active in IUGR/FGR placentas. Upregulation of mitochondrial chaperones and proteases, such as CLPP, was seen in several studies, indicating an attempt to reduce mitochondrial stress levels. However, in extreme situations, the UPRmt response may be inadequate, resulting in unresolved mitochondrial dysfunction and further limiting placental capacity. Notably, comparisons between symmetric and asymmetric FGR indicated that symmetric instances, which are often associated with earlier start and more severe placental dysfunction, had higher mtDNA depletion, lower ATP levels, and more severe oxidative damage than asymmetric cases. In monochorionic twin pregnancies exacerbated by selective FGR, molecular investigations revealed mitochondrial abnormalities exclusive to the placenta supporting the growth-restricted twin, reinforcing the direct relationship between placental mitochondrial health and fetal growth potential.

Overall, our data show that mitochondrial dysfunction in IUGR/FGR is caused by a complex interaction of reduced mitochondrial biogenesis, faulty oxidative phosphorylation, excessive oxidative stress, unbalanced mitochondrial dynamics, and poor mitochondrial quality control. These molecular abnormalities combine to limit trophoblast proliferation, syncytialization, and nutrient transport processes, resulting in reduced fetal development.

#### 3.3.3. Preterm Birth

Placental mitochondrial dysfunction has also been linked to the pathophysiology of PTB; however, the molecular processes appear to differ from those seen in PE and FGR. Several studies included in this review found mitochondrial alterations in placentas from spontaneous PTB cases, even in the absence of classical PE features or significant fetal growth restriction, implying that mitochondrial dysfunction may contribute independently to the risk of premature parturition. At the molecular level, PTB placentas had fewer mtDNA copies than term pregnancies. This discovery suggests a reduction in mitochondrial mass or defective biogenesis, which might restrict the placenta’s energy-generating capabilities. Decreased ATP generation was also seen, but less consistently and severely than in PE or IUGR patients, indicating mild but physiologically significant bioenergetic impairment.

Oxidative stress has emerged as a key molecular characteristic in PTB-associated placental disease. Several studies found higher ROS levels and lipid peroxidation products, including MDA. Oxidative stress in the placenta can activate inflammatory signaling pathways, including the nuclear factor-kappa B (NF-κB) pathway, resulting in the generation of pro-inflammatory cytokines including interleukin-6 (IL-6) and tumor necrosis factor-alpha (TNF-α). This inflammatory environment is known to promote cervical ripening, membrane rupture, and myometrial activation, all of which play important roles in the onset of spontaneous labor.

In addition to oxidative stress, mitochondrial dynamics were discovered to be changed in PTB patients. An imbalance favoring mitochondrial fission over fusion has been identified, as evidenced by increased DRP1 and decreased MFN2. Excessive mitochondrial fragmentation may make trophoblast cells susceptible to apoptosis and reduce mitochondrial energy efficiency, contributing to placental malfunction. Furthermore, mitochondrial fragmentation has been demonstrated to activate inflammatory pathways and augment innate immunological responses, suggesting a possible mechanistic relationship between mitochondrial changes and the premature activation of parturition cascades.

Disruptions to mitochondrial quality-control systems were also observed. Reduced expression of PINK1 and PARK2 in PTB placentas indicates poor mitophagy and the buildup of defective mitochondria. Failure to efficiently remove damaged mitochondria may increase ROS production and activate inflammasomes, such as the nucleotide-binding oligomerization domain-like receptor protein 3 (NLRP3) inflammasome, exacerbating inflammatory reactions.

While mitochondrial dysfunction in PTB was less severe than in PE or severe FGR, the combined effect of mild mitochondrial damage, oxidative stress, and inflammation appeared to be sufficient to disrupt placental homeostasis and trigger preterm delivery. These findings lend credence to a paradigm in which mitochondrial failure is both a source of oxidative stress and a trigger for inflammatory pathways that result in spontaneous PTB.

In summary, the data from subgroup analyses show that placental mitochondrial dysfunction plays a significant role in the pathophysiology of severe neonatal outcomes. While mitochondrial abnormalities such as mtDNA content reductions, impaired ATP production, elevated ROS generation, disrupted mitochondrial dynamics, and defective quality-control mechanisms were consistently observed in PE, IUGR/FGR, and PTB, the severity and specific molecular signatures differed between clinical phenotypes. Early-onset PE and symmetric FGR showed the most substantial mitochondrial abnormalities, indicating a main role for placental malfunction, whereas mitochondrial disruptions in PTB appeared to contribute indirectly via oxidative stress and inflammatory pathways. These findings demonstrate the critical role of mitochondrial integrity in placental function and embryonic development. They also lend credence to the concept that tailored therapies focused at protecting mitochondrial health might be effective techniques for preventing or improving unfavorable perinatal outcomes. The next section contains a full description of these results, their putative causes, and therapeutic implications.

## 4. Discussion

### 4.1. Summary of Principal Findings

This comprehensive review emphasizes the importance of placental mitochondrial dysfunction in the pathogenesis of key unfavorable prenatal outcomes such as PE, IUGR, FGR, and PTB. The data consistently show that changes in mitochondrial biogenesis, energy generation, oxidative stress management, mitochondrial dynamics, and quality-control pathways contribute to the development of these problems. Across the studies considered, mitochondrial dysfunction was linked to reduced mtDNA maintenance, decreased ATP synthesis, increased ROS production, and deficient mitochondrial turnover, all of which compromised placental homeostasis and fetal growth potential. In particular, the severity and molecular features of mitochondrial dysfunction vary depending on the clinical phenotype. Early-onset PE and symmetric FGR were linked to the most severe mitochondrial abnormalities, indicating that primary placental failure plays a prominent role in these diseases. In contrast, mitochondrial anomalies reported in PTB appeared to promote oxidative stress and inflammatory signaling pathways, resulting in preterm labor rather than direct placental insufficiency.

These findings support the notion that mitochondrial health is required for proper placental growth and function. They further imply that mitochondrial dysfunction may act as both a biomarker of placental impairment and a molecular driver of disease development. Understanding the molecular mechanisms behind mitochondrial changes in complex pregnancies might lead to novel pathways for early identification, risk stratification, and targeted treatment therapies aimed at preserving mitochondrial function and enhancing neonatal outcomes.

Placental insufficiency, characterized by the placenta’s inadequate support of fetal growth due to structural or functional deficiencies, is increasingly recognized as being partially influenced by mitochondrial malfunction. Common features of placental insufficiency, disruptions in mitochondrial energy production, biogenesis, and redox equilibrium can restrict trophoblast invasion, vascular remodeling, and nutrient delivery. The findings of our review suggest that mitochondrial dysfunctions precede and mechanistically contribute to the emergence of clinical conditions such as preeclampsia, fetal growth restriction, and preterm birth, positioning mitochondrial impairment not only as a molecular correlate but also as a potential initiating factor in the sequence of placental failure.

### 4.2. Mechanistic Insights into Placental Mitochondrial Dysfunction

According to Xiang-Qun Hu and Lubo Zhang (2022), mitochondrial dysfunction in preeclampsia (PE) is closely connected to impaired electron transport and the excessive formation of reactive oxygen species (ROS), resulting in oxidative stress and vascular dysfunction [21]. This conclusion is consistent with the discoveries made by Sanchez-Aranguren et al. (2018) [24], who found lower ATP generation, higher ROS levels, and mitochondrial DNA (mtDNA) damage in preeclamptic placentas. Both studies agree on the importance of mitochondrial bioenergetic failure in the pathophysiology of PE [24]. Yucheng Hu et al. (2024) [35] extended on their prior findings by offering more detailed mechanistic insights into selective fetal growth restriction (sFGR). In addition to confirming mitochondrial morphological abnormalities in cytotrophoblasts, elevated ROS levels, reduced ATP production, and reduced mtDNA integrity, their recent analysis revealed an increase in mtDNA copy number associated with heterozygous mutations in mitochondrial ribosomal genes. Furthermore, they discovered dysregulation of mitochondrial long non-coding RNAs (lncND5, lncND6, and lncCytb), indicating a new layer of epigenetic control that contributes to mitochondrial dysfunction in sFGR. These comprehensive molecular findings complement and expand on the findings presented in our work, which identified diminished mtDNA integrity and increased oxidative stress as the main causes of selective FGR [35].

Reinaldo Marín et al. (2020) found changes in mitochondrial dynamics between early-onset preeclampsia (eoPE) and late-onset preeclampsia (loPE), including the differential expression of fission and fusion mediators including dynamin-related protein 1 (DRP1) and mitofusins (MFN1/2) [57]. These findings are consistent with those published by Ausman et al. (2018) and Mathyk et al. (2018), who found increased DRP1 and reduced mitofusin-2 in PE placentas, demonstrating that mitochondrial fragmentation and defective fusion are molecular characteristics of placental insufficiency [27,28]. Venkata Ramana Vaka et al. (2018) provided additional data from the reduced uterine perfusion pressure (RUPP) model showing higher mitochondrial ROS contribute directly to maternal hypertension via mitochondrial malfunction in endothelial and placental cells [58]. This molecular cascade is consistent with the findings of Deer et al. (2020) in Table 4, which show that angiotensin II type 1 autoantibodies (AT1-AA)-mediated oxidative stress is linked to vascular mitochondrial damage in PE [33].

Wu et al. (2024) examined mitochondrial quality control mechanisms, especially mitophagy and unfolded protein response (UPRmt), and concluded that the failure of these systems might result in trophoblast cell malfunction [59]. This review supports the findings of Yung et al. (2019) and Suksai et al. (2024) in Table 4, which show impaired UPRmt signaling and elevated mitochondrial nuclear retrograde regulator 1 (MNRR1), respectively, linking defective mitochondrial clearance and stress signaling to preeclamptic pathology [43,45].

Fahmida Jahan et al. (2023) demonstrated that mitochondrial dysfunction is a unifying mechanism across various clinical subtypes of PE [4], supporting the findings of Zhang et al. (2022), who reported increased DRP1 phosphorylation and necroptotic signaling in trophoblasts, emphasizing mitochondrial-driven cell death pathways in PE progression [26]. Cristina Mandò et al. (2013) found that while IUGR placental tissues have more mtDNA, cytotrophoblasts have less mitochondrial functioning [60]. This seeming contradiction is mirrored in the findings of Lattuada et al. (2008), suggesting that mitochondrial biogenesis may be a compensatory but ultimately inadequate response to mitochondrial oxidative stress in IUGR [37]. Finally, Philippe Vangrieken et al. (2021) [46] found that PE placentas had lower mitochondrial biogenesis indicators, improved mitophagy machinery, and elevated apoptosis and inflammation, correlating with the results in Table 4. These findings emphasize the link between mitochondrial metabolic dysfunction and inflammatory pathways in the disease’s pathophysiology [46].

### 4.3. Differential Mitochondrial Patterns Across Pregnancy Complications

Although placental mitochondrial dysfunction is a common factor in poor pregnancy outcomes, there are significant differences in the molecular characteristics and severity of mitochondrial impairment across clinical phenotypes such as preeclampsia (PE), intrauterine growth restriction (IUGR)/fetal growth restriction (FGR), and preterm birth (PTB) [4].

In preeclampsia, particularly in early-onset PE (eoPE), mitochondrial dysfunction appears to be severe and complex. Marín et al. (2020) found increased mitochondrial fission in eoPE placentas, characterized by overexpression of DRP1 and downregulation of MFN1 and MFN2, resulting in fragmented mitochondrial networks [57]. These findings are comparable with those reported by Sanchez-Aranguren et al. (2018) and Mathyk et al. (2018), who found structural mitochondrial abnormalities and higher ROS production in PE patients [24,28]. Marín et al. (2020) showed decreased complex IV activity and poor oxidative phosphorylation, indicating substantial bioenergetic failure [57]. Philippe Vangrieken et al. (2021) found a concurrent drop in mitochondrial biogenesis indicators and increased mitophagy in PE, supporting the idea of overburdened mitochondrial quality-control systems [46]. In contrast, mitochondrial changes in late-onset PE (loPE) are rather moderate. Marín et al. (2020) [57] discovered that while some mitochondrial dysfunctions continue in loPE, such as moderate decreases in oxidative phosphorylation efficiency, the level of fission/fusion imbalance and ROS overproduction is far less severe than in eoPE. These findings show that mitochondrial dysfunction in loPE may work as an adaptive reaction rather than a fundamental cause of illness [57].

Mitochondrial dysfunction in IUGR and FGR presents predominantly as decreased biogenesis and reduced energy output, albeit with subtle differences. Cristina Mandò et al. (2013) [60] found that IUGR placentas had higher mtDNA concentration in tissues but lower expression of mtDNA and NRF1 in isolated cytotrophoblasts. This contradiction suggests a compartment-specific compensation mechanism aimed at maintaining ATP generation during hypoxic stress [60]. Similarly, Lattuada et al. (2008) found lower oxygen consumption and respiratory efficiency in cytotrophoblasts from IUGR pregnancies (Table 4) [37]. Yucheng Hu et al. (2024) added an important dimension by identifying mitochondrial genomic mutations and dysregulated long non-coding RNAs (lncND5, lncND6, and lncCytb) in selective FGR placentas, implying that genetic and epigenetic mitochondrial alterations play critical roles in driving fetal growth restriction [35].

Interestingly, mitochondrial oxidative stress in IUGR/FGR is slightly less severe than in PE. Although elevated ROS levels and oxidative damage indicators such as malondialdehyde (MDA) were frequently seen (Hu et al., 2024 [35]; Guitart-Mampel et al., 2019 [61]), the amount of mitochondrial complex dysfunction was often less severe than in PE. This divergence may help to explain why IUGR/FGR is frequently characterized by persistent placental insufficiency but lacks the significant systemic maternal symptoms associated with PE [35,61].

Mitochondrial malfunction has a unique role in preterm birth (PTB). The available evidence, including findings from Wu et al. (2024) and data evaluated in Table 4 by Zhou et al. (2017), indicates that placental mitochondrial abnormalities in PTB are mild, but they are adequate to initiate inflammatory cascades [54,59]. In the RUPP model, Venkata Ramana Vaka et al. (2018) [58] found that mitochondrial oxidative stress increases the levels of inflammatory mediators including TNF-α and IL-6, leading to premature labor onset. Unlike in PE and IUGR, where direct bioenergetic failure is predominant, mitochondrial ROS in PTB largely operate as inflammatory triggers, hastening membrane rupture and uterine contractions [58].

Thus, whereas mitochondrial dysfunction is a common underlying illness, the molecular profile differs.

- Early-onset PE is characterized by significant mitochondrial fragmentation, impaired oxidative phosphorylation, and overwhelmed quality control.
- In IUGR/FGR, mitochondrial abnormalities include impaired biogenesis, mild-to-moderate ROS buildup, and adaptive bioenergetic responses at the cytotrophoblast level.
- In PTB, mitochondrial stress largely enhances inflammatory signaling pathways, resulting in premature birth.

These diverse patterns not only highlight pathophysiological disparities but also imply that future treatment methods may need to be tailored to the individual mitochondrial disease found in each pregnancy problem.

### 4.4. Potential Clinical Implications and Therapeutic Perspectives

The recognition of placental mitochondrial dysfunction as a key mechanism in bad neonatal outcomes has significant therapeutic implications [62]. Understanding the distinct mitochondrial signatures associated with preeclampsia (PE), intrauterine growth restriction (IUGR), fetal growth restriction (FGR), and preterm birth (PTB) provides new opportunities for early detection, risk stratification, and targeted therapies to improve maternal and fetal outcomes [63]. One possible therapeutic use is the use of mitochondrial biomarkers in early identification and prognosis. Hu et al. (2024) and Mandò et al. (2013) found that mtDNA content and quality changes precede clinical indications of placental malfunction [35,60]. Cowell et al. (2021) proposed measuring circulating cell-free mtDNA in maternal blood as a non-invasive biomarker for the early prediction of PE and FGR [32]. Furthermore, the study of mitochondrial-derived peptides and long non-coding RNAs (such as lncND5 and lncCytb discovered by Hu et al., 2024) may give molecular signs of placental stress even before overt clinical indications appear [35].

Another clinical implication comes from the discovery that increased mitochondrial reactive oxygen species (mtROS) generation is harmful in PE and PTB [64]. In the RUPP model, Vaka et al. (2018) found that treating mitochondrial oxidative stress with particular antioxidants like MitoQ and MitoTEMPO dramatically decreased maternal hypertension while improving placental mitochondrial respiration [58]. This proof-of-concept study demonstrates that mitochondria-targeted antioxidant therapy might be a useful technique for reducing placental oxidative damage and its systemic effects. Coenzyme Q10 supplementation has been proven to improve mitochondrial electron transport chain activity and reduce oxidative stress in PE patients (Marín et al., 2020), validating this treatment strategy [57].

Beyond antioxidants, therapies aimed at restoring mitochondrial quality-control systems are gaining popularity. Wu et al. (2024) [59] and Vangrieken et al. (2021) [46] found that defective mitophagy worsens placental mitochondrial damage. Therapies that enhance PINK1–PARK2-mediated mitophagy, whether by pharmacological stimulation or gene therapy, have the potential to selectively remove defective mitochondria while retaining trophoblast function and avoiding placental insufficiency. Although yet experimental, targeted manipulation of mitochondrial turnover represents a promising area in prenatal medicine [46,59].

Furthermore, modification of mitochondrial dynamics may be investigated as a therapeutic target. Marín et al. (2020) [57] and Mathyk et al. (2018) [28] found that imbalanced mitochondrial fission and fusion causes trophoblast dysfunction in PE. Mdivi-1, an inhibitor of DRP1-mediated mitochondrial fission, has been proven in preclinical models to reduce mitochondrial fragmentation and oxidative damage. Their use in pregnancy problems requires careful evaluation, especially with regard to fetal safety. Interventions must take into account disease heterogeneity [28,57]. As Jahan et al. (2023) [4] point out, not all PE or FGR patients show the same level or kind of mitochondrial dysfunction. Personalized approaches, guided by mitochondrial biomarkers or placental imaging modalities that assess mitochondrial function (e.g., mitochondrial-targeted fluorescent imaging agents), may allow for patient stratification for tailored therapies, maximizing efficacy while reducing unnecessary interventions [4].

The developmental origins of health and disease (DOHaD) approach emphasizes the need for early mitochondrial treatments. Guitart-Mampel et al. (2019) [61] found that mitochondrial dysfunction that occurs during fetal life remains postnatally, predisposing children to cardiovascular and metabolic illnesses. As a result, medicines that improve placental mitochondrial activity may improve not just immediate neonatal outcomes but also provide long-term health advantages to future generations [61]. Despite these enticing prospects, some problems must be addressed. The timing, specificity, and safety profile of mitochondrial-targeted treatments during pregnancy must be thoroughly evaluated [65]. Furthermore, standardization of mitochondrial tests and the construction of rigorous reference ranges for mitochondrial biomarkers are required prior to clinical translation.

### 4.5. Strengths and Limitations of the Present Review

This systematic analysis assesses the involvement of placental mitochondrial dysfunction in unfavorable perinatal outcomes, including PE, IUGR/FGR, and PTB. A crucial strength is careful adherence to PRISMA principles, which ensure transparency and repeatability. The thorough database search and stringent qualifying criteria enabled the inclusion of high-quality research concentrating on placental mitochondrial changes. One of the review’s key features is its focus on particular mitochondrial metrics such as mtDNA integrity, ATP production, ROS generation, mitochondrial dynamics (DRP1, MFN1, and MFN2), and mitochondrial quality control mechanisms (PINK1, PARK2, and UPRmt). By concentrating on these molecular elements, this review gives a complete mechanistic understanding of how mitochondrial dysfunction contributes to the pathology of PE, IUGR/FGR, and PTB.

Furthermore, this research thoroughly contrasted data across pregnancy problems, emphasizing distinct mitochondrial patterns such as severe ETC impairment and increased mitophagy activation in PE vs. adaptive biogenesis responses in IUGR/FGR. The use of evidence from both clinical and translational investigations improves the validity of the results reached. However, many restrictions should be recognized. Although strict eligibility criteria were used, the inherent heterogeneity of study designs, sample types (whole placental tissue vs. isolated trophoblasts), and methodologies for assessing mtDNA, ATP, ROS, and mitochondrial dynamics introduces variability that may limit the generalizability of the findings. Differences in gestational age during sample collection, as well as different definitions of PE and IUGR, may further make comparisons difficult.

Another drawback is the lack of research on new mitochondrial indicators, such as mitochondrial lncRNAs, mitophagy-related proteins other than PINK1/PARK2, and comprehensive profiling of UPRmt components. Furthermore, while the current review focused on human trials to improve therapeutic relevance, essential molecular insights gained from animal models were excluded. Finally, while prospective therapeutic targets have been discovered, the translational usefulness of therapies like mitochondrial antioxidants or mitophagy modulators requires additional confirmation in large-scale human studies. Overall, despite these limitations, the current study increases our understanding of mitochondrial dysfunction in pregnancy problems and emphasizes the importance of future standardized, mechanistically focused research.

## 5. Future Research Directions

Building on the growing evidence of placental mitochondrial dysfunction in unfavorable pregnancy outcomes, many essential avenues for future research appear. Addressing present knowledge gaps will be critical for translating mitochondrial biology into therapeutically relevant applications. First, large-scale, multicenter prospective cohort studies are urgently required to study mitochondrial changes across pregnancy problems. These investigations should involve systematic measurements of mtDNA quantity and quality, ATP levels, ROS generation, and mitochondrial dynamics (DRP1, MFN1, and MFN2) in order to create accurate reference ranges and identify pathological thresholds. Uniform techniques, such as standardized tissue sampling, uniform gestational age at collection, and validated mitochondrial function tests, are required to allow for cross-study comparisons and strong meta-analyses.

Second, further investigation of mitochondrial epigenetics is a potential field. Although Hu et al. (2024) [35] discovered the involvement of mitochondrial lncRNAs (lncND5, lncND6, and lncCytb), their mechanistic role in mitochondrial homeostasis, bioenergetics, and oxidative stress responses is still poorly understood. Future research should look at how epigenetic changes within the mitochondrial genome lead to placental malfunction, and if these signals might be used as early prognostic biomarkers in maternal blood or other non-invasive matrices [35].

Third, technical advances in mitochondrial imaging provide interesting diagnostic opportunities. The discovery and practical use of mitochondrial-targeted fluorescent imaging agents, along with sophisticated microscopy or non-invasive imaging systems, may allow for the real-time monitoring of placental mitochondrial activity in vivo. Such techniques would enable the early detection of pregnancies at risk of PE, IUGR/FGR, or PTB before irreparable placental damage occurs. In addition, expanding therapeutic research is crucial. Although mitochondrial antioxidants such as MitoQ and MitoTEMPO have demonstrated efficacy in lowering mtROS and improving outcomes in preclinical models (Vaka et al., 2018), clinical trials evaluating their safety, appropriate dose, and effectiveness in pregnant women are limited [58]. Future research should look into combinatorial therapeutics that target several mitochondrial pathways, such as ROS scavenging, ETC stability, and ATP production.

Beyond antioxidant methods, regulating mitochondrial dynamics and quality control offers a potential therapeutic approach. Pharmacological inhibitors of DRP1-mediated fission (such as Mdivi-1) or mitophagy activators via PINK1–PARK2 pathways have the potential to restore mitochondrial network integrity and remove damaged mitochondria, hence preserving trophoblast survival and functionality. However, before practical translation, fetal safety and off-target consequences must be thoroughly evaluated. Furthermore, studies should look at the effects of fetal sex on mitochondrial adaptability and disease manifestation. Hebert and Myatt (2021) [18] found that placental mitochondrial responses to stresses such as oxidative damage may be sexually dimorphic, with male and female fetuses demonstrating unique sensitivity patterns. Stratified analyses and sex-specific therapy techniques may improve treatment effectiveness and perinatal outcomes [18].

Longitudinal follow-up investigations into the effects of placental mitochondrial dysfunction on child health trajectories are also critical. Guitart-Mampel et al. (2019) [61] found chronic mitochondrial bioenergetic deficits in neonates born from IUGR pregnancies, implying programming effects that last into adolescence and adulthood. Future studies should combine prenatal mitochondrial evaluation with long-term cardiovascular, metabolic, and neurodevelopmental monitoring to better understand the developmental origins of health and disease (DOHaD) paradigm [61]. Finally, new omics tools like mitochondrial proteomics, metabolomics, and transcriptomics should be used to offer complete assessments of placental mitochondrial status. Multi-omics integration is expected to identify novel biomarkers and therapeutic targets that single-parameter analysis would miss.

To summarize, future research on mitochondrial biology in pregnancy must take a multidisciplinary, multidimensional approach, combining clinical, molecular, imaging, and systems biology techniques. Only via such collaborative efforts will mitochondrial findings be translated into concrete therapeutic advancements, therefore benefiting the health of mothers and children.

## 6. Conclusions

The current comprehensive analysis emphasizes the critical role of placental mitochondrial dysfunction in the etiology of significant unfavorable pregnancy outcomes, including as PE, IUGR/FGR, and PTB. Specific mitochondrial alterations, such as reduced mtDNA integrity, impaired ATP synthesis, excessive ROS production, dysregulated mitochondrial dynamics involving DRP1, MFN1, and MFN2, and defective quality control mechanisms via the PINK1, PARK2, and UPRmt pathways, are critical contributors to placental insufficiency, fetal growth restriction, hypertensive disorders of pregnancy, and premature labor according to accumulating evidence. Importantly, diverse mitochondrial patterns were found across pregnancy problems. Early-onset PE is characterized by severe oxidative phosphorylation disruption, widespread mitochondrial fragmentation, and overwhelming mitophagy. IUGR/FGR exhibits adaptive mitochondrial biogenesis and mild oxidative stress, whereas PTB’s mitochondrial failure largely stimulates inflammatory signaling rather than energy depletion pathways. These different mitochondrial fingerprints underline the importance of developing diagnostic and treatment solutions tailored to each problem.

Mitochondrial biomarkers, including as cell-free mtDNA, mitochondrial-derived peptides, and mitochondrial lncRNAs, are potential techniques for the early identification of placental malfunction. Therapeutic strategies that target mtROS, restore ETC function, improve mitophagy, and modulate mitochondrial dynamics are emerging as novel ways to slow the onset of pregnancy problems. However, clinical translation necessitates comprehensive evaluation of these techniques’ effectiveness, safety, and the timing of the intervention. Furthermore, the long-term effects of intrauterine mitochondrial dysfunction on child health, as indicated by the DOHaD hypothesis, highlight the need to accelerate research efforts to preserve mitochondrial integrity throughout pregnancy. To further understand and improve prenatal outcomes, future research should use multi-omics techniques, sex-specific analysis, longitudinal follow-up, and standardized mitochondrial functional tests.

In conclusion, placental mitochondrial dysfunction is an important and changeable factor of mother and fetal health. Targeting mitochondrial health provides a revolutionary potential to not only alleviate current pregnancy difficulties but also favorably affect future generations’ health trajectories.

## Figures and Tables

**Table 1:** Shows the search strategy used for each database.

Database	Search Terms Used
**PubMed**	(“placenta” [MeSH Terms] OR “placenta” [All Fields]) AND (“mitochondrial dysfunction” [All Fields] OR “mitochondria” [MeSH Terms] OR “mitochondria” [All Fields]) AND (“oxidative stress” [MeSH Terms] OR “oxidative stress” [All Fields]) AND (“preeclampsia” [MeSH Terms] OR “preeclampsia” [All Fields] OR “intrauterine growth restriction” [MeSH Terms] OR “intrauterine growth restriction” [All Fields] OR “preterm birth” [MeSH Terms] OR “preterm birth” [All Fields] OR “stillbirth” [MeSH Terms] OR “stillbirth” [All Fields])
**Scopus**	TITLE-ABS-KEY (placenta) AND TITLE-ABS-KEY (mitochondrial dysfunction OR mitochondria) AND TITLE-ABS-KEY (oxidative stress) AND TITLE-ABS-KEY (preeclampsia OR intrauterine growth restriction OR preterm birth OR stillbirth)
**Web of Science**	TS = (placenta) AND TS = (mitochondrial dysfunction OR mitochondria) AND TS = (oxidative stress) AND TS = (preeclampsia OR intrauterine growth restriction OR preterm birth OR stillbirth)

Abbreviations: TS, topic search; MeSH, medical subject heading.

**Table 2:** Summary of main mitochondrial findings in placental tissues associated with adverse perinatal outcomes.

Study (First Author, Year)	Outcome	Mitochondrial Findings	Main Conclusion
Sanchez-Aranguren et al., 2018 [24]	PE	Decreased ATP, increased ROS, and mtDNA damage	Mitochondrial dysfunction contributes to PE
Huang et al., 2021 [25]	PE	Decreased RND3 expression and mitochondrial impairment	RND3 deficiency leads to placental mitochondrial dysfunction
Zhang et al., 2022 [26]	PE	Increased PGAM5 and p-DRP1-S637	Induction of mitochondrial fission and necroptosis
Ausman et al., 2018 [27]	PE	Increased DRP1 and decreased OPA1	Mitochondrial fragmentation linked to PE
Mathyk et al., 2018 [28]	PE	Decreased Mitofusin-2 expression	Impaired mitochondrial fusion in PE
Beyramzadeh et al., 2017 [29]	PE, PTB, FGR	Reduced ETC complex activities	Placental energy deficiency
Bînă et al., 2022 [30]	PE	Increased monoamine oxidase activity	Enhanced oxidative stress in PE placentas
Broady et al., 2017 [31]	PE	Increased oxidative stress markers (8-OHdG) and decreased SIRT1/3	Mitochondrial oxidative damage in PE
Cowell et al., 2021 [32]	PTB, low birth weight	Increased mtDNA mutational load	Placental mitochondrial dysfunction linked to PTB
Deer et al., 2020 [33]	PE	Increased AT1-AA-mediated oxidative stress	Mitochondrial-stress-response activation
Holland et al., 2018 [34]	PE	Decreased DRP1 and altered mitochondrial structure	Mitochondrial adaptation failure associated with PE
Hu et al., 2024 [35]	FGR	Decreased mtDNA and increased oxidative stress	Mitochondrial dysfunction associated with selective FGR
Kiyokoba et al., 2022 [36]	PE, FGR	Increased GDF15 expression	Mitochondrial stress signaling involved
Lattuada et al., 2008 [37]	FGR	Increased mtDNA content	Possible compensatory response to mitochondrial stress
Lefebvre et al., 2018 [38]	PE, FGR	Decreased mitochondrial respiration	Mitochondrial insufficiency in placental insufficiency
Marschalek et al., 2018 [39]	PE	Elevated circulating mtDNA	Biomarker of placental mitochondrial dysfunction
Meng et al., 2020 [40]	FGR (MCDA twins)	Altered miRNA expression (miR-199a)	miRNA dysregulation linked to mitochondrial dysfunction
Naha et al., 2020 [41]	FGR	mtDNA mutations and decreased copy number	Mitochondrial genome instability associated with FGR
Pandey et al., 2020 [42]	Early-onset PE	Reduced mtDNA copy number	Biomarker potential for early PE detection
Yung et al., 2019 [43]	Early-onset PE	Impaired UPRmt and decreased oxidative phosphorylation	UPRmt failure contributes to PE
Shi et al., 2013 [44]	Hypertensive disorders	Defective mitochondrial FAO pathways	Placental mitochondrial dysfunction associated with hypertension
Suksai et al., 2024 [45]	PE	Increased MNRR1 expression	Mitochondrial stress biomarker elevation in PE
Vangrieken et al., 2021 [46]	PE	Decreased mitochondrial biogenesis markers	Mitochondrial content reduction in PE placentas
Vishnyakova et al., 2016 [47]	Early- and late-onset PE	Altered OPA1 expression	Mitochondrial dynamics disruption
Vishnyakova et al., 2017 [48]	Early- and late-onset PE	Impaired antioxidant enzymes (SOD (Superoxide Dismutase), and Catalase)	Increased oxidative damage in PE
Wang et al., 1998 [49]	PE	Elevated MDA levels	Oxidative stress mediated placental dysfunction
Williamson et al., 2018 [50]	PE	Decreased mtDNA copy number	Evidence of mitochondrial impairment
Williamson et al., 2019 [51]	PE	TLR9 activation	Inflammatory mitochondrial response in PE
Xu et al., 2021 [52]	FGR	Decreased mtDNA content and altered mitochondrial proteins	Placental mitochondrial compromise linked to FGR
Yu et al., 2016 [53]	PE	Downregulation of Mitofusin-2	Impaired mitochondrial fusion associated with PE
Zhou et al., 2017 [54]	PE	Altered mitochondrial morphology and lipid metabolism	Mitochondrial dysfunction linked to placental pathology
Xu et al., 2018 [55]	Early-onset PE	Impaired mitochondrial morphology and biogenesis	Mitochondrial defects drive PE development
Zsengellér et al., 2016 [56]	PE	Reduced COX activity	Mitochondrial respiratory chain impairment

Abbreviations: PE, preeclampsia; PTB, preterm birth; FGR, fetal growth restriction; ROS, reactive oxygen species; ATP, adenosine triphosphate; mtDNA, mitochondrial DNA; ETC, electron transport chain; DRP1, dynamin-related protein 1; OPA1, optic atrophy 1; SIRT, sirtuin; UPRmt, mitochondrial unfolded protein response; Malondialdehyde, MDA; GDF15, growth differentiation factor; Toll-like receptor 9; COX, cytochrome c oxidase; FAO, fatty acid oxidation; and MCDA, monochorionic diamniotic.

**Table 3:** Risk of bias assessment of the included studies using the Newcastle–Ottawa scale.

Study (First Author, Year)	Selection	Comparability	Outcome/Exposure	Overall Risk of Bias
Sanchez-Aranguren et al., 2018 [24]	Good	Good	Good	Low
Huang et al., 2021 [25]	Good	Moderate	Good	Moderate
Zhang et al., 2022 [26]	Moderate	Moderate	Good	Moderate
Ausman et al., 2018 [27]	Good	Good	Good	Low
Mathyk et al., 2018 [28]	Good	Moderate	Good	Moderate
Beyramzadeh et al., 2017 [29]	Moderate	Moderate	Moderate	Moderate
Bînă et al., 2022 [30]	Moderate	Moderate	Good	Moderate
Broady et al., 2017 [31]	Good	Moderate	Good	Moderate
Cowell et al., 2021 [32]	Good	Good	Good	Low
Deer et al., 2020 [33]	Moderate	Moderate	Moderate	Moderate
Holland et al., 2018 [34]	Good	Good	Good	Low
Hu et al., 2024 [35]	Moderate	Moderate	Moderate	Moderate
Kiyokoba et al., 2022 [36]	Moderate	Moderate	Good	Moderate
Lattuada et al., 2008 [37]	Good	Moderate	Good	Moderate
Lefebvre et al., 2018 [38]	Good	Good	Good	Low
Marschalek et al., 2018 [39]	Good	Moderate	Good	Moderate
Meng et al., 2020 [40]	Moderate	Moderate	Moderate	Moderate
Naha et al., 2020 [41]	Moderate	Moderate	Good	Moderate
Pandey et al., 2020 [42]	Moderate	Moderate	Good	Moderate
Yung et al., 2019 [43]	Moderate	Moderate	Good	Moderate
Shi et al., 2013 [44]	Good	Moderate	Moderate	Moderate
Suksai et al., 2024 [45]	Good	Good	Good	Low
Vangrieken et al., 2021 [46]	Moderate	Moderate	Good	Moderate
Vishnyakova et al., 2016 [47]	Moderate	Moderate	Moderate	Moderate
Vishnyakova et al., 2017 [48]	Moderate	Moderate	Moderate	Moderate
Wang et al., 1998 [49]	Moderate	Moderate	Moderate	Moderate
Williamson et al., 2018 [50]	Good	Moderate	Good	Moderate
Williamson et al., 2019 [51]	Good	Moderate	Good	Moderate
Xu et al., 2021 [52]	Moderate	Moderate	Good	Moderate
Yu et al., 2016 [53]	Moderate	Moderate	Good	Moderate
Zhou et al., 2017 [54]	Moderate	Moderate	Good	Moderate
Xu et al., 2018 [55]	Moderate	Moderate	Moderate	Moderate
Zsengellér et al., 2016 [56]	Good	Moderate	Good	Moderate

Each included study’s risk of bias was examined using the Newcastle–Ottawa scale, which evaluated three domains, study group selection, group comparability, and outcome/exposure assessment. Methodological quality was used to assign “Good”, “Moderate”, or “Poor” ratings to each domain. The total risk of bias for each study was classified as low, moderate, or high based on the cumulative quality of the evaluated domains.

**Table 4:** Summarizes the main mitochondrial findings in placental tissues associated with worse perinatal outcomes.

Year	Author	Type of Study	Outcome Investigated	Mitochondrial Parameter Implicated
**2018**	Sanchez-Aranguren et al. [24]	Case–control study	Preeclampsia	mtDNA
**2021**	Huang et al. [25]	Case–control study	Preeclampsia	Rnd3
**2022**	Zhang et al. [26]	Case–control study	Preeclampsia	PGAM5 and p-DRP1-S637
**2018**	Ausman et al. [27]	Case–control study	Preeclampsia	DRP1, OPA1
**2018**	Mathyk et al. [28]	Cross-sectional study	Preeclampsia	Mitofusin-2
**2017**	Beyramzadeh et al. [29]	Case–control study	Preeclampsia, PTB, and FGR	ETC complexes I-IV and citrate synthase
**2022**	Bînă et al. [30]	Case–control study	Preeclampsia	Monoamine oxidase
**2017**	Broady et al. [31]	Case–control study	Preeclampsia	visfatin/Nampt, SIRT1, SIRT3, and 8OHdG
**2021**	Cowell et al. [32]	Prospective cohort	PTB and birth weight	mtDNA
**2020**	Deer et al. [33]	Case–control study	Preeclampsia	AT1-AA
**2018**	Holland et al. [34]	Case–control study	Preeclampsia	Dynamin-related Protein 1 (DRP1), OPA1, CASP3, and Glutathione Peroxidase (GPx)
**2024**	Hu et al. [35]	Prospective cohort	Selective FGR	mtDNA
**2022**	Kiyokoba et al. [36]	Case–control study	Preeclampsia and FGR	GDF15
**2008**	Lattuada et al. [37]	Case–control study	FGR	mtDNA
**2018**	Lefebvre et al. [38]	Case–control study	Preeclampsia and FGR	mtDNA
**2018**	Marschalek et al. [39]	Case–control study	Preeclampsia	mtDNA
**2020**	Meng et al. [40]	Case–control study	Selective FGR	miRNA
**2020**	Naha et al. [41]	Case–control study	FGR	mtDNA
**2019**	Pandey et al. [42]	Case–control study	Early-onset preeclampsia	mtDNA
**2013**	Shi et al. [44]	Case–control study	HDP	FAO, HADHA, HADHB, and ACADVL
**2024**	Suksai et al. [45]	Case–control study	Preeclampsia	MNRR1
**2021**	Vangrieken et al. [46]	Case–control study	Preeclampsia	PGC-1α, PGC-1β, NRF2α, BNIP3, PARK2, etc.
**2016**	Vishnyakova et al. [47]	Case–control study	Early- and late-onset preeclampsia	OPA1
**2017**	Vishnyakova et al. [48]	Case–control study	Early- and late-onset preeclampsia	SOD1, SOD2, Catalase, PGC-1α, etc.
**1998**	Wang et al. [49]	Case–control study	Preeclampsia	MDA
**2018**	Williamson et al. [50]	Case–control study	Preeclampsia	SOD and mtDNA
**2019**	Williamson et al. [51]	Case–control study	Preeclampsia	TLR9
**2021**	Xu et al. [52]	Case–control study	FGR	mtDNA, ND6, COX I/IV, and VDAC
**2016**	Yu et al. [53]	Case–control study	Preeclampsia	Mitofusin-2
**2017**	Zhou et al. [54]	Case–control study	Preeclampsia	ATP, lipids, and amino acid derivatives
**2018**	Xu et al. [55]	Case–control study	Early-onset preeclampsia	Morphology, PRDX2, BCL2, etc.
**2016**	Zsengellér et al. [56]	Case–control study	Preeclampsia	COX

The methodological characteristics of the included studies. Abbreviations: **GA**: gestational age, **mtDNA**: mitochondrial DNA, **PGAM5**: phosphoglycerate mutase 5, **AT1-AA**: angiotensin II type I receptor, **DMN1L/DRP1**: pro-fission dynamin-1-like protein, **CASP3**: cleaved caspase 3, **GPx**: glutathione peroxidase, **UPRmt**: mitochondrial unfolded protein response, **CLPP**: quality-control protease in UPRmt signaling, **FAO**: fattyacid oxidation, **HADHA**: hydroxyacyl-Coenzyme A dehydrogenase alpha subunit, **miRNA**: microRNA, **HADHB**: hydroxyacyl-Coenzyme A dehydrogenase beta subunit, **ACADVL**: acyl-Coenzyme A dehydrogenase, very-long chain, **MNRR1**: mitochondrial nuclear retrograde regulator 1, **PGC-1**: peroxisome proliferator-activated receptor gamma coactivator 1, **NRF2α**: nuclear respiratory factor 1, **BNIP3**: BCL2/adenovirus E1B 19 k Da, protein-interacting protein 3, **BNIP3L**: BCL2/adenovirus E1B 19 kDa protein-interacting protein 3-like, **FUNDC1**: FUN14 domain containing 1, **PINK1**: PTEN-induced kinase 1, **PARK2**: E3 ubiquitin-protein ligase Parkin, **DNM1L**: Dynamin-related protein 1, **Fis-1**: fission 1 protein, **OPA1**: optic atrophy protein 1, **SOD1**: cytoplasmic superoxide dismutase 1, **SOD2**: mitochondrial superoxide dismutase 2, **GPx1**: glutathione peroxidase 1, **TLR9:** toll-like receptor 9, **8OHdG**: 8-oxo-deoxyguanosine, **SIRT**: sirtuin, **DMN1L/DRP1**: pro-fission dynamin-1-like protein, **ND6:** NADH dehydrogenase 6, **COX I:** cytochrome *c* oxidase I, **COX IV:** respiratory chain complex IV, **VDAC:** mitochondrial membrane proteins, **HIF-1α:** mitophagy receptor, **PRDX2:** peroxiredoxin 2, **PARK7:** parkinsonism-associated deglycase, **SUCLG1:** alpha subunit of the succinyl-CoA, **ACADM**: acyl-CoA dehydrogenase, **HDP:** hypertensive disorders of pregnancy, **DM**: diabetes mellitus, **GDM**: gestational diabetes, **PE**: preeclampsia, **DCDA:** dichorionic, diamniotic twin pregnancy, **MCDA**: monochorionic diamniotic twin pregnancy, **OCP**: oral contraceptive pills, **IUD**: intrauterine death, **SCD**: sickle cell disease, **SLE**: systemic erythematous lupus, **STD**: sexually transmitted disease, **PROM**: preterm rupture of membranes, **FGR**: fetal growth restriction, and **ICP**: intrahepatic cholestasis.
